# Development of a patient decision aid for type 2 diabetes mellitus: a patient-centered approach

**DOI:** 10.1186/s12875-025-02772-7

**Published:** 2025-03-22

**Authors:** Anna Tichler, Dorijn F. L. Hertroijs, Dirk Ruwaard, Martijn C. G. J. Brouwers, Arianne M. J. Elissen

**Affiliations:** 1 Department of Health Services Research, Care and Public Health Research Institute (CAPHRI), Faculty of Health, Medicine and Life Sciences (FHML), Maastricht University, Maastricht, the Netherlands; 2 Care and Public Health Research Institute (CAPHRI), Faculty of Health, Medicine and Life Sciences (FHML), Maastricht University, Maastricht, the Netherlands; 3 Department of Internal Medicine, Division of Endocrinology and Metabolic Disease, Maastricht University Medical Center+, Maastricht, the Netherlands

**Keywords:** Person-centered care, Shared decision-making, Patient decision aid, Type 2 diabetes mellitus, Primary care

## Abstract

**Background:**

Patient decision aids (PDAs) can effectively facilitate shared decision-making (SDM) between patients and healthcare professionals. The International Patient Decision Aid Standards (IPDAS) Collaboration created a model for the systematic development of PDAs. However, the absence of a solid evidence base limits practical recommendations for best practices. For example, uncertainties exist about the most appropriate method for identifying the needs and preferences of patients and healthcare professionals. This study aims to detail the development process for the development of a PDA for type 2 diabetes mellitus (T2DM), using the IPDAS model.

**Methods:**

From September 2020 to February 2023, we systematically developed the PDA for T2DM in the Netherlands. We adopted a patient-centered approach by researching patient considerations and actively collaborating with a multidisciplinary steering group, including patients with T2DM, patient organizations, and healthcare professionals. The PDA content and prototype development were determined by incorporating patients’ needs and preferences, input from the steering group, and available evidence regarding T2DM treatment options. The research team and steering group iteratively reviewed the PDA prototype.

**Results:**

A web-based PDA was developed consisting of five sections: 1) information about T2DM and the available treatment options; 2) comparison of treatment options; 3) questions to assess patients’ knowledge; 4) value-clarification exercise; and 5) summary of the patient’s journey through the PDA. Before patients use the PDA, healthcare professionals can preselect the most relevant treatment options.

**Conclusions:**

Early and iterative involvement of relevant stakeholders in the development process of the PDA helped the alignment of the PDA with the needs and preferences of the diverse end-users. In a future study, we will investigate the effectiveness of the PDA in facilitating SDM in T2DM care.

**Trial registration:**

International Clinical Trials Registry Platform ID: NL8948, date of registration: 05–10-2020.

**Supplementary Information:**

The online version contains supplementary material available at 10.1186/s12875-025-02772-7.

## Background

Person-centered care emphasizes the importance of patients’ active involvement in treatment decisions [1]. Shared decision-making (SDM) is a person-centered approach that empowers patients to play an active role in the treatment decision-making process and is used to reach the best treatment decision for patients [2–4]. In the SDM process, healthcare professionals and patients engage in a deliberative dialogue about possible treatment options and their risks and benefits, and consider patients’ values, needs, and preferences in the decision-making process [5]. For this process to work effectively, it is key that the patient and healthcare professional work together to arrive at the best possible treatment decision [3]. Decision-making is particularly complex in cases of preference-sensitive conditions, where there are multiple medically accepted options available. Hence, the best treatment choice should be based on the patient’s preferences and values [6].

Patient decision aids (PDAs) are useful tools to support patients and healthcare professionals in effectively engaging in SDM [7]. PDAs provide information on the decision that needs to be made and all the available treatment options, including their risks and benefits. Moreover, PDAs can help patients clarify their personal values and preferences regarding these treatment options. Over the last twenty years, interest in PDAs has been growing [7, 8]. A wide range of PDAs has been developed to address many different treatment decisions around conditions such as breast cancer, osteoarthritis, and chronic kidney disease [9]. A systematic review by Stacey et al. [10] showed that the use of PDAs to facilitate SDM led to better-informed patients with more accurate expectations of the risks and benefits of treatment options, higher satisfaction about both their decision and the quality of the decision-making process and increased confidence in the decision-making process compared to patients who did not use a PDA. Furthermore, the use of PDAs can have a positive effect on patient-clinician communication.

In response to the growing use of PDAs, the International Patient Decision Aid Standards (IPDAS) Collaboration developed a model for the systematic development of PDAs, which was subsequently updated to incorporate user-centered design principles and methods [11, 12]. This model serves as a guide for the developers of PDAs, aims to improve the feasibility and acceptability of PDAs in practice, as well as to provide a comprehensive overview of the development process of PDAs. The IPDAS model includes the following steps: 1) scoping; 2) establishment of a steering group; 3) content and format (i.e., assessment of patients’ and healthcare professionals' needs and preferences, format and distribution plan, and review and synthesize evidence); 4) prototype development; 5) alpha testing and 6) beta testing (i.e., ‘real life’ testing with patients and healthcare professionals not involved in the development process). The IPDAS Collaboration also emphasizes the involvement of patients and healthcare professionals throughout the development process. However, practical guidance is limited due to the absence of a solid evidence base to support best practices [13]. For example, the most suitable approach for defining decisional needs is unknown, and questions remain on how to best involve patients and healthcare professionals in the development process. Moreover, there is a lack of agreement on the selection of information for inclusion in the PDA [14].

This paper aims to provide practical insights into using the IPDAS systematic development process (up and until step 5) for the development of a PDA for type 2 diabetes mellitus (T2DM), a condition where patient preferences play a crucial role. Several PDAs for T2DM have been developed and evaluated worldwide [15]. These PDAs have different formats (paper-based and digital) and focus on various treatment decisions, such as insulin initiation and medication intensification. However, no PDA specifically tailored to the needs of patients with T2DM currently exists to enhance person-centered care through SDM. Adopting a patient-centered approach, this PDA was developed through extensive research into patient considerations and active collaboration with both patients and healthcare professionals during the development process. The PDA is intended for adults with T2DM to use independently in between consultations, as preparation for making a treatment decision with their healthcare professional. The paper provides a comprehensive overview of the development process, including key stakeholders and methodologies employed.

## Methods

**Fig. 1:**
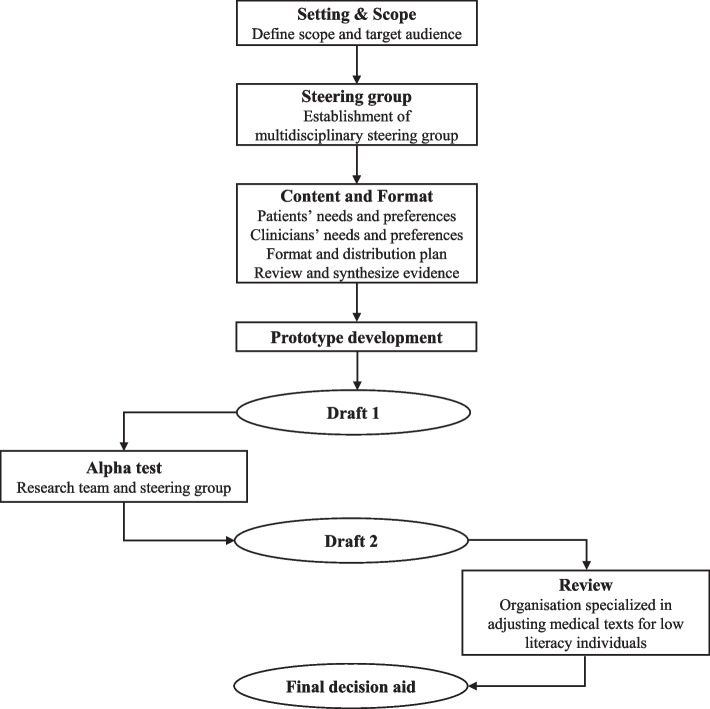
The systematic development process of the patient decision aid for type 2 diabetes mellitus

The PDA for T2DM was systematically developed in the Netherlands between September 2020 and February 2023. An overview of the development process for the PDA can be found in Fig. 1. The study as part of the development of the PDA for T2DM was approved by the Dutch Clinical Research Foundation (NWMO20.03.015) and by the Medical Ethics Review Committee of the academic hospital of Maastricht (azM) and Maastricht University (020–2176). The study was conducted in agreement with the ethical standards described in the Declaration of Helsinki. Written informed consent was obtained from all participants.

### Setting and scope

In the Netherlands, the majority of patients with T2DM (89% in 2021) receive diabetes care in a primary care setting [16]. General practitioners have responsibility for most aspects of T2DM care. In practice, most diabetes care is carried out by practice nurses or trained diabetes nurses [17]. The general practitioner conducts annual assessments for patients with T2DM to detect and manage risk factors and complications associated with the condition. Treatment is provided following the national guidelines on T2DM of the Dutch College of General Practitioners (NHG) [18]. In recent years, new pharmacological agents (e.g., sodium-glucose cotransport 2 [SGLT2] inhibitors and glucagon-like peptide 1 [GLP-1] receptor agonists) have been introduced in the Standards of Medical Care in Diabetes (the American Diabetes Association’s [ADA] standards) and NHG guidelines for high-risk patients with T2DM [18, 19]. Patients are considered high risk when they have a history of cardiovascular diseases or heart failure. Multiple treatment paths are now possible, resulting in an increasingly complex decision-making process for patients and their healthcare professionals. Therefore, a PDA for T2DM can provide significant benefits. The PDA aims to help both patients and their healthcare professionals in navigating the complex treatment decisions in primary care. The target users are adults with T2DM who need to decide on a new or additional treatment, which can involve lifestyle change and/or medical options. The PDA can be used by patients in between consultations to help them learn more about their condition, treatment options, and personal preferences. Once completed, the PDA can guide the decision-making process during the consultations with their healthcare professional. The PDA is meant to be complementary to the consultation where the treatment decision is made.

### Establishment of a steering group

The PDA was developed in the Netherlands in close collaboration with relevant stakeholders in Dutch diabetes care. The development process was coordinated by a multidisciplinary research team (*N* = 5) with expertise in person-centered care and medical expertise in T2DM. A steering group was established at the beginning of the development process (*N* = 11), consisting of patients with T2DM (*N* = 2), a representative from the Dutch Diabetes Association (in Dutch: Diabetes Vereniging Nederland, DVN), a representative from the Netherlands Diabetes Federation (in Dutch: Nederlandse Diabetes Federatie, NDF), a general practitioner (also representing the Dutch College of General Practitioners), a diabetes nurse, two practice nurses, a pharmacist, an endocrinologist, and a dietician. The steering group participated in the development of the PDA at the level of partnership [20]. There was a high level of engagement and collaboration between the research team and steering group, as well as equal decision-making power. Within the steering group, relevant experts in the field of diabetes care could share their knowledge and opinions on the development of the PDA. This helped in aligning the PDA as much as possible to the diverse needs of the end-users. Moreover, involving the relevant stakeholders throughout the development process is essential to facilitate the broad implementation of the PDA in practice. The steering group met twice a year (online) and advised on the development process and content of the PDA. The meetings lasted approximately two hours. The steering group meetings were interactive, and small-group discussions were held using statements about different aspects of the PDA, such as the format. Members of the steering group also reviewed the content of the PDA several times.

### Content and format

After establishing the steering group, we followed a four-step design (based on the IDPAS guidelines) to gather relevant information in preparation for the PDA prototype development. First, we conducted a needs assessment to identify and prioritize the attributes that patients with T2DM want to discuss with their healthcare professional in the decision-making process. Second, the steering group provided their perspectives on the needs assessment of patients and their own preferences. Third, based on these findings, the steering group and research team decided on the format of the PDA for T2DM. Finally, evidence relevant to T2DM treatment options was reviewed and synthesized. Each of these steps is described in detail below.

First, we started with an exploratory sequential design to assess patients’ needs and preferences. A detailed description of this study is described elsewhere [21]. In short, the aim was to identify attributes (i.e., conversation topics) that patients with T2DM want to discuss with their healthcare professionals in the decision-making process. Three small group interviews and one in-depth interview were held, including a total of 8 patients with T2DM, recruited using a convenience sampling method. During the small group interviews, a list of 21 attributes was identified that were valued as important in the treatment decision-making process. The attributes were subsequently prioritized from most to least important using a best–worst scaling survey, with 285 participants with a completed survey included in the analysis. This survey was used to determine the importance of each identified conversation topic relative to all other conversation topics [22]. ‘Quality of life’ was valued as most important in the decision-making process for the treatment of T2DM, followed by ‘clinical outcomes’, ‘long-term diabetes complications’, ‘short-term adverse events of medication’, and ‘lifestyle’. The full list of attributes can be found in the article of Tichler et al. [21]. Second, members of the steering group provided their perspectives on the evidence-based needs assessment of the patients with T2DM and expressed their own needs and preferences. The steering group mostly agreed with the results of the patient’s needs assessment. Third, informed by the patient’s needs and preferences, the research team and steering group decided to move forward with the development of an online web-based PDA. This PDA format was preferred due to its multipurpose, offering various advantages (e.g., patients can access the information at home and additional information can be found via hyperlinks to reliable sources). For the development of an online web-based PDA, we established a partnership with PatientPlus, which is the largest developer and supplier of PDAs in the Netherlands [23]. Finally, evidence relevant to the treatment decision and options were reviewed and synthesized, taking into account the NHG treatment guidelines on T2DM [18]. This included the extraction of evidence from systematic reviews, scientific articles, and guidelines on the effectiveness and safety of T2DM treatment options, as well as evidence on side effects from the Dutch Healthcare Institute (Farmacotherapeutisch Kompas) [24]. Information was selected based on outcomes that were found important by patients and healthcare professionals (i.e., results of needs assessment), the Dutch Diabetes Association, the Netherlands Diabetes Federation, and PatientPlus. This information was complemented by the expertise of healthcare professionals from the research team and steering group. The PDA includes a list of referenced scientific literature and provides information on the last revision date. Following the IPDAS guidelines, the content of the PDA will be subjected to regular review and updating every two years by PatientPlus [11].

### Prototype development and alpha testing

The PDA development was initiated by presenting a summarized content outline to the steering group (see Supplementary File 1). This one-page summary included key information, such as the target group and treatment options, and listed the crucial components to be included in the PDA. Following agreement on the outline, a scientific writer of PatientPlus started the development of a paper prototype of the PDA incorporating all relevant evidence. An online infrastructure was simultaneously built within the existing platform of PatientPlus (www.keuzehulp.info).

The PDA prototype was iteratively reviewed and revised by the research team and steering group. First, each member of the research team provided individual feedback on the PDA’s content, which was then processed by PatientPlus. Second, the steering group participants individually reviewed the PDA and their feedback was also processed by PatientPlus. In the third step, the steering group members verified if their input was adequately addressed. This resulted in multiple revisions of the PDA. In the fourth step, the final version of the PDA prototype was presented to the research team and steering group for consensus. Fifth, upon achieving consensus, the PDA was assessed and adjusted by an organization specialized in adjusting medical texts for low-literacy individuals (in Dutch: Stichting Makkelijk Lezen) [25]. Finally, the PDA for T2DM was added to the online catalog of PatientPlus.

While the alpha testing phase included iterative feedback and revisions involving the research team, steering group, and an organization specialized in low-literacy adaptations, formal usability testing was not conducted at this stage. Future work will include usability testing to systematically evaluate the PDA’s user experience, comprehensibility, and accessibility among patients and healthcare professionals.

### IPDAS assessment

The quality of the final PDA prototype was assessed using the quality criteria checklist of IPDAS Collaboration [7, 26]. The checklist consists of 64 criteria in three domains: content, development process and effectiveness. The effectiveness of the PDA is not assessed in this study and there are no patient stories (examples of experiences of others that are relevant to the decision that needs to be made) included in the PDA. Therefore, 45 of the 64 quality criteria were applicable. Two researchers individually evaluated the quality of the PDA using the IPDAS checklist, whereafter consensus was reached in a meeting.

## Results

**Fig. 2:**
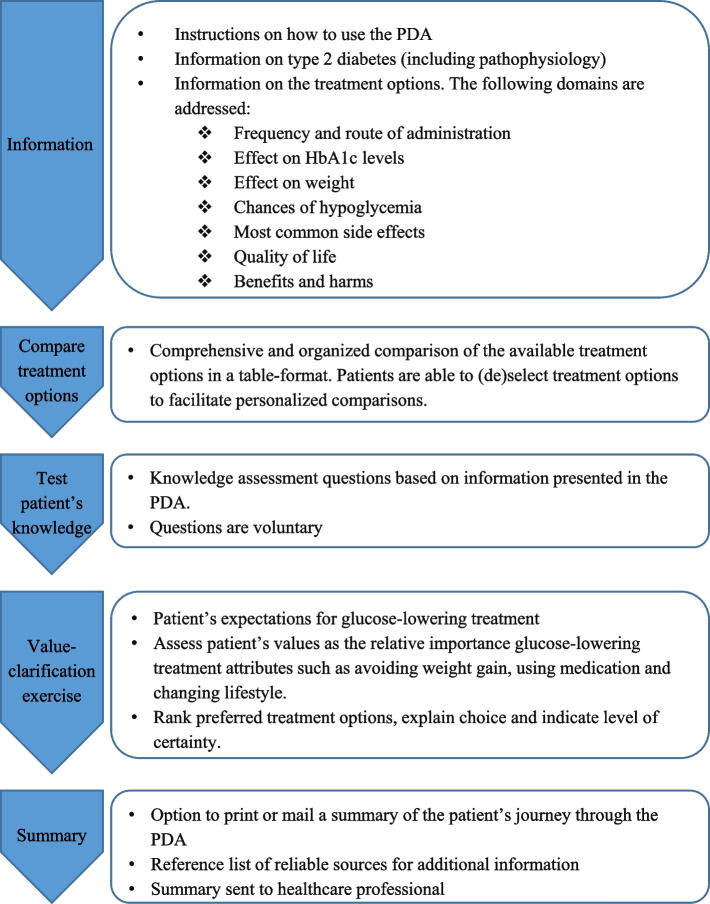
Overview of the content of the patient decision aid (PDA) for type 2 diabetes mellitus

The web-based PDA for T2DM is available in the online catalog of PatientPlus (https://www.keuzehulp.info/front-page/keuzehulpen/diabetes-type-2, Dutch only). The PDA received the quality mark ‘common language’ from an organization specialized in adjusting medical text for low-literacy individuals [25]. This means the PDA is easy to read and understandable. In line with IPDAS guidance, the PDA is structured into five sections, consistent with all PatientPlus PDAs: 1) information about T2DM and the different treatment options; 2) comparison of treatment options; 3) questions to test patient’s knowledge; 4) value-clarification exercise; and 5) summary of the patient’s answers and notes. The content of each section is presented in Fig. 2.

The first section provides instructions on how to use the decision aid and information about T2DM, including a summarized and simplified pathophysiology of T2DM. Moreover, the different treatment options for T2DM are explained. The treatment options included in the PDA are no treatment, lifestyle changes, metformin, sulfonylureas, dipeptidyl peptidase-4 (DPP-4) inhibitors, glucagon-like peptide 1 (GLP-1) receptor agonists, sodium-glucose cotransport 2 (SGLT2) inhibitors and insulin. However, due to individual characteristics (and reimbursement restrictions), not all treatment options are suitable for every patient. Therefore, healthcare professionals select relevant options before the patient uses the PDA. In the first two sections of the PDA, the patient receives information on these selected options, with ‘no treatment’ and ‘lifestyle changes’ always included. It is a standard feature of all PatientPlus PDAs to include the ‘no treatment’ option, meeting a criterion in the IPDAS quality framework [7]. The ‘lifestyle change’ option is included as a standard option since patients considered the ‘lifestyle’ attribute an important conversation topic in the T2DM treatment decision-making process [21]. Furthermore, the steering group wanted to emphasize the importance of lifestyle changes in treating T2DM.

For each treatment option, information is provided on its characteristics, such as frequency and route of administration (for medical treatment options). The most important conversation topics in the T2DM treatment decision-making process, as valued by patients [21], have also been incorporated in this section of the PDA. This includes quality of life, clinical outcomes, long-term diabetes complications, and short-term adverse events of medication. Both positive and negative impacts of each treatment on a patient’s quality of life is provided. For example, metformin may improve quality of life by reducing diabetes symptoms and lowering the risk of cardiovascular disease but may also have negative effects such as side effects and the need to take pills daily. The information on clinical outcomes is divided into the treatment’s effect on HbA1c levels and weight. Short-term adverse events of medication are separated into the risk of hypoglycemia and the most common side effects of each medication type. The information on long-term diabetes complications is divided into the risk of cardiovascular disease and other micro- and macrovascular complications that may occur as a consequence of T2DM. All the aforementioned information is summarized into a short list categorized into the benefits and harms of each treatment option.

The second section of the PDA presents a comprehensive and organized comparison of the available treatment options in a table format. The table presents a summary of each treatment, including the treatment’s characteristics (i.e., frequency and route of administration), effect on HbA1c levels and weight, long-term diabetes complications (including risk of cardiovascular disease), risk of hypoglycemia, most common side effects, and benefits and harms. The patient can select and deselect treatments and their characteristics for a personalized comparison.

The third section consists of knowledge assessment questions (*N* = 9) based on the information presented in the PDA. These multiple-choice questions are included to assess whether patients correctly understood the information presented in the PDA. For example, patients will be asked the following question: ‘Which medication increases the risk of hypoglycemia?’. If the patient is uncertain of the answer, they are recommended to read the relevant information in Sections 1 and 2 of the PDA again or discuss it with their healthcare professional. The patient has the option to skip the questions.

The fourth section of the PDA includes a value-clarification exercise to support patients in determining their values and preferences related to their glucose-lowering treatment. This section also incorporates the attribute ‘quality of life’ and is divided into three parts. In the first part, patients are asked to share their treatment expectations. This includes describing what quality of life means to them and how they would like to see their quality of life improve during or after treatment. Second, the patient’s values and preferences are assessed using statements on several attributes, such as avoiding weight gain, using medication, changing lifestyle, and avoiding adverse events of medication. For example, patients are presented with choices such as ‘I want to lose weight’, ‘I do not mind if my weight increases’, or ‘I have no opinion’, with corresponding treatment options provided. In the third part, patients are asked to rank their preferred treatment options, explain their choice for the most preferred option, and indicate their level of certainty. Additionally, they can write down any remaining questions or concerns for their healthcare professional. Throughout the PDA, patients can take notes, which will be saved for them.

The final section enables patients to print, download, or mail a summary, which includes their answers to all the questions and notes. The summary can also be sent to their healthcare professional. Furthermore, patients can find a list of reliable sources for additional information on T2DM and the treatment options (e.g. the website of the Dutch Diabetes Association). The PDA explicitly does not provide advice for a particular treatment. It aims to help patients and healthcare professionals to decide on the most suitable treatment.

### IPDAS assessment

The checklist of the IPDAS Collaboration was used to assess the quality of the final PDA prototype. A total of 45 of the 64 quality criteria were applicable to our study. The final PDA prototype met 33 out of the 45 applicable criteria (73%) (Supplementary Table 1). The criteria that were not met primarily concerned the presentation of probabilities of outcomes, as there are no definitive numerical values for treatment outcomes in the T2DM context. Although the PDA does not present exact probabilities, it does provide relevant information such as the average HbA1c reduction for each treatment.

## Discussion

In this paper, we described in detail the development of a web-based PDA for T2DM using the IPDAS systematic development process model and following a patient-centered approach. The PDA was developed in close collaboration with a steering group representing all relevant stakeholders in Dutch diabetes care (e.g. patients with T2DM, patient organizations, and healthcare professionals). We started with extensive research into patients’ needs and preferences in the treatment decision-making process. These preferences, in combination with input from the steering group and the available evidence on the treatment options for T2DM, were used to determine the content of the PDA and to develop a web-based PDA. The PDA consists of five sections: 1) information about T2DM and the different treatment options; 2) comparison of treatment options based on, for example, clinical outcomes, risk of cardiovascular diseases, and effect on daily life; 3) questions to test patients’ knowledge; 4) value-clarification exercise; and 5) summary of the patient’s answers and notes.

Several PDAs have been developed and tested in different countries specifically for the treatment of T2DM [15]. For example, the Diabetes Medication Choice decision aid, developed in the US, is for patients who are not (yet) using insulin and have multiple treatment options to consider [27]. This PDA compares metformin, insulin, GLP-1 agonists, sulfonylureas, DPP-4 inhibitors, SGLT2 inhibitors, and thiazolidinediones. Similarly, the PANDA paper-based PDA, developed in the UK, is for patients who need to consider changing their current T2DM treatment to insulin therapy [28]. The PDA includes treatment options for no change, lifestyle change, and insulin therapy. A final example is the Diabetes Decision Aid, developed in the US, and designed for patients taking metformin with persistent hyperglycemia who need to consider medication intensification [29]. This PDA compares sulfonylureas, DPP-4 inhibitors, thiazolidinediones, SGLT2 inhibitors, GLP-1 agonists, and insulin.

In comparison, our PDA has several unique features. First, it encompasses all treatment options outlined in national and international guidelines for T2DM care (lifestyle change and medical treatment options) [18, 19]. It covers each possible treatment decision, including the option of no treatment. Second, healthcare professionals can make a preselection of the most relevant treatment options prior to the patient using the PDA. Considering the many treatment options available, this unique feature is important to reduce the complexity of the decision-making process for both patients with T2DM and their healthcare professionals. Third, our PDA contains components tailored specifically for patients with T2DM in the Netherlands, addressing their unique needs and preferences. For example, information on treatment costs was only added as additional reading material (through a drop-down) because it was not valued as important by Dutch patients [21]. This may be due to different healthcare systems across countries, which influences whether treatment for T2DM is covered by health insurance, potentially impacting the need for information on this topic [30]. While our PDA contains a distinct set of features, it also shares similarities. Specifically, the PANDA PDA and our PDA include information on the impact of a treatment on a patient’s quality of life and a value-clarification exercise. The methods employed during the development might have resulted in the unique features of our PDA, as well as similarities with the PANDA PDA. Both PDAs were developed using the IPDAS systematic development process model and following a patient-centered approach by extensively researching the needs and preferences of patients, in contrast with the Diabetes Medication Choice decision aid and Diabetes Decision Aid. The patient-centered approach allowed us to better understand patients’ needs and tailor the PDA accordingly. We recommend following the IPDAS model and adopting a patient-centered approach for the development of a PDA for preference-sensitive conditions.

The digital format of the PDA offers various advantages. Patients can access and review the information at home, the PDA includes hyperlinks to reliable sources, providing patients with the option to access additional, in-depth, information about diabetes and its treatment and the PDA can be personalized since healthcare professionals can preselect treatment options. Additionally, the digital PDA offers the opportunity for rapid and effortless adjustments to its content, as PatientPlus takes responsibility for regular reviews and updates (at least every two years). The digital format also enables the future collection of valuable information on the usage behavior of patients (e.g., the duration of usage from start to end), which could inform iterative improvements to the PDA. While the digital format of the PDA offers various advantages, it is important to also acknowledge its limitations. In general, research has shown that adults aged 60 years and older have lower levels of digital literacy compared to individuals aged below 60 years [31, 32]. The average age of diagnosis for T2DM in the Netherlands was 60.9 years in 2019, suggesting lower digital literacy among patients with T2DM [33]. This lower level of digital literacy, as well as negative attitudes toward and a lack of trust in health technology, poses barriers to adopting health technology among older adults [34, 35]. Stacey et al. [36] found that online PDAs can hinder their use in clinical practice post-trials, and Doll et al. [37] reported that approximately half of the patients felt uncomfortable using a tablet device and instead preferred the paper-based PDA for coronary artery disease. While people may face challenges with using online PDAs, there are effective ways to address these barriers, such as general education and support from healthcare professionals [34]. Ultimately, the advantages of an online PDA format, including the possibility to access and review the information at home and the option to preselect treatment options, make it a valuable tool.

Patients have different preferences regarding the amount of information they would like to receive when making a treatment decision, as some prefer more detailed information than others [38]. Previous research has also highlighted the importance of patients receiving information that is accurate, up-to-date, and relevant [39]. Unclear or overwhelming information can hinder patients from using the information effectively to make informed decisions. To address these concerns, our PDA enables healthcare professionals to make a preselection for suitable treatment options based on a patient’s clinical aspects. Informing patients about all the treatment options is a quality criterion of the IPDAS [7]. However, to prevent overwhelming patients with unnecessary and irrelevant information, and because the T2DM treatment guidelines provide a stepped-care protocol (which means that not all treatment options are suitable for every patient at specific time points), we opted not to provide patients with information about all the treatment options. Furthermore, for interested patients, our PDA includes additional reading material via drop-down menus. This includes, for example, information on the mechanism of action for medical treatment options. Moreover, a list of useful and reliable websites is provided.

The development process of the PDA for T2DM has some strengths and limitations. We adopted a patient-centered approach and involved relevant stakeholders in diabetes care, which aligns with one of the recommendations for effective PDA implementation of Joseph-Williams et al. [8]. The co-creation with relevant stakeholders improves the quality of the PDA and can be helpful for successful implementation in clinical practice. Moreover, the PDA is evidence-based and aligned with the national treatment guidelines. While collaborating with multiple stakeholders has various advantages, it is important to consider some of the challenges that arise in the process. For example, the iterative review of the PDA’s content can make the development process long. Moreover, it has been challenging to integrate all the diverse views of the stakeholders and find a consensus. We organized an additional meeting with some stakeholders to facilitate dialogue and reach a consensus on specific aspects of the PDA’s content. Facilitators of the development process included early and consistent stakeholder engagement, which helped to build trust and align shared goals. Furthermore, the IPDAS framework provided a structured roadmap for the PDA development process. Based on our experience, we recommend allocating sufficient time for stakeholder involvement and iterative revisions. It is also important to give patients sufficient opportunities to share their input during joint meetings with healthcare professionals. Clearly outlining the expected level of engagement from stakeholders at the onset of PDA development can further improve the process. These strategies can help address potential barriers and maximize the benefits of involving patients and healthcare professionals in the development of PDAs.

## Conclusion

We adopted a patient-centered approach for the development of a web-based PDA for T2DM (https://www.keuzehulp.info/front-page/keuzehulpen/diabetes-type-2, Dutch only) through extensive research into patient needs, preferences and considerations, and active collaboration with patients and healthcare professionals during the development process. Involving patients and healthcare professionals in an early and iterative way throughout the development process is valuable for ensuring that the PDA is as compatible as possible with the diverse needs and preferences of all relevant stakeholders in Dutch diabetes care. Following the IPDAS model, the final PDA prototype will be field-tested in a ‘real-life’ setting with patients and healthcare professionals who were not involved in the development process. Currently, a pilot study is being conducted to improve the quality and feasibility of a subsequent full-scale economic evaluation (ZonMw project number: 10390052210053). The pilot study focuses on questions related to recruitment and retention, study management, and feasibility of outcome measurement. The full economic evaluation will assess the PDA’s (cost-)effectiveness in primary care, focusing on short-term SDM outcomes and long-term outcomes. Short-term SDM outcomes include decisional conflict, level of SDM, and patient knowledge. Long-term outcomes include quality of life, treatment adherence, costs from a societal perspective, and glycemic control. The results of these studies will guide refinements to enhance the PDA’s usability and ensure effective implementation into routine practice. Based on these findings, primary care practices may consider using the PDA as a supporting tool in the T2DM treatment decision-making process. Additionally, training HCPs in SDM and the use of the PDA may be necessary to maximize the tool’s effectiveness. Overall, the tool has the potential to facilitate SDM and guide person-centered care in the treatment of T2DM in primary care.

## Supplementary Information

Supplementary Material 1.

Supplementary Material 2.

## Data Availability

Data sharing is not applicable to this article as no datasets were generated or analyzed during the current study.

## Abbreviations

ADA: American Diabetes Association
DPP-4: Dipeptidyl peptidase-4
DVN: Diabetesvereniging Nederland (Dutch Diabetes Association)
EASD: European Association for the Study of Diabetes
GLP-1: Glucagon-like peptide 1
IPDAS: International Patient Decision Aid Standards
NDF: Nederlandse Diabetes Federatie (Netherlands Diabetes Federation)
NHG: Nederlands Huisartsen Genootschap (Dutch College of General Practitioners)
PDA: Patient Decision Aid
SDM: Shared Decision-Making
SGLT2: Sodium-glucose cotransport 2
T2DM: Type 2 Diabetes Mellitus
UK: United Kingdom
US: United States
