# Supplementary material for: Development of a patient decision aid for type 2 diabetes mellitus: a patient-centered approach
Source: BMC Prim Care. 2025 Mar 22;26:81. doi: 10.1186/s12875-025-02772-7 (PMC11929313; doi:10.1186/s12875-025-02772-7)
Supplement: Supplementary file 1 — Supplementary Material 1. [file 12875_2025_2772_MOESM1_ESM.docx]

**Additional File 1. One-page summary of the patient decision aid**

| **Topic** | **Type 2 Diabetes Mellitus: lifestyle and medical treatment** |
| --- | --- |
| Target audience | Adults with type 2 diabetes mellitus |
| Introduction | - How can the patient decision aid help you? - What is type 2 diabetes mellitus? (Including links to reliable websites where people can find more information?) - Causes - Symptoms - Long-term complications (including the risk of cardiovascular diseases) - Quality of life - Treatment (including monitoring and explanation of hypoglycemia) |
| Treatment options | - No treatment - Lifestyle modification - Metformin - GLP-1 receptor agonist - DPP4-inhibitor - SGLT2-inhibitor - Sulfonylurea derivatives (SU- derivatives) - Insulin |
| Explanation of each treatment option | - What does the treatment entail? - How does the treatment work? - Impact on daily life - For whom is the treatment suitable? |
| Outcomes for each treatment | - How effective is it?   - Blood sugar levels: will I achieve good blood sugar levels?   - Weight: will my weight change?   - Quality of life   - Risk of cardiovascular disease - Risks   - Adverse events of treatment   - Hypoglycemia: risk of hypoglycemia?   - Long-term effects: what are the long-term effects? - Advantages - Disadvantages |
| Test your knowledge | - Can I combine lifestyle changes with medication? - Which medications can cause hypoglycemia? - Do I need to administer the medication through injections? - Will I lose weight from the treatment? - Will the side effects of the medical treatment diminish if I continue using it? - Quality of life |
| What is important to you? | - Weight - Administration (injections/oral) - Effort required (lifestyle modification) - Self-monitoring blood sugar levels (insulin) - Self-adjusting dosage (insulin) - Already tried certain options (e.g. losing weight) on my own - Hypoglycemia - Adverse events (general) |
| Developed by | PatientPlus and Maastricht University Medical Centre+ (MUMC+) in collaboration with patients with type 2 diabetes (N=2), a representative from the Dutch Diabetes Association, a representative from the Netherlands Diabetes Federation, a representative from the Dutch College of General Practitioners (also a general practitioner), a pharmacist, practice nurses (N=2), a diabetes nurse, a medical specialist and a dietician. |
| Supported by | Dutch Diabetes Association (in Dutch: Diabetesvereniging Nederland, DVN), the Netherlands Diabetes Federation (in Dutch: Nederlandse Diabetes Federatie, NDF) and the Dutch College of General Practitioners (in Dutch: Nederlands Huisartsen Genootschap, NHG) |
