# Supplementary material for: Development of a patient decision aid for type 2 diabetes mellitus: a patient-centered approach
Source: BMC Prim Care. 2025 Mar 22;26:81. doi: 10.1186/s12875-025-02772-7 (PMC11929313; doi:10.1186/s12875-025-02772-7)
Supplement: Supplementary file 2 — Supplementary Material 2. [file 12875_2025_2772_MOESM2_ESM.docx]

**Supplementary Table S1**

**International Patient Decision Aids Standards (IPDAS) checklist to assess quality**

Table 1. The International Patient Decision Aids Standards (IPDAS) checklist to assess the quality of the PDA for T2DM.

| **Criteria** | **Meet criteria?** |
| --- | --- |
| **Domain 1. Content: does the patient decision aid…** | |
| **Provide information about the options in sufficient detail for decision-making?** | |
| 1. Describe the health condition | Yes |
| 1. List the options | Yes |
| 1. List the option of doing nothing | Yes |
| 1. Describe the natural course without options | Yes |
| 1. Describe procedures | Yes |
| 1. Describe positive features (benefits) | Yes |
| 1. Describe negative features of options (harms/side effects/disadvantages) | Yes |
| 1. Include chances of positive/negative outcomes | No |
| *Additional items for tests* | |
| 1. Describe what test is designed to measure | NA |
| 1. Include chances of true positive, true negative, false positive, false negative test results | NA |
| 1. Describe possible next steps based on test results | NA |
| 1. Include chances the disease is found with/without screening | NA |
| 1. Describe detection/treatment that would never have caused problems if one was not screened | NA |
| **Present probabilities of outcomes in an unbiased and understandable way?** | |
| 1. Use event rates specifying the population and time period | No |
| 1. Compare outcome probabilities using the same denominator, time period, scale | No |
| 1. Describe uncertainty around probabilities | No |
| 1. Use visual diagrams | No |
| 1. Use multiple methods to view probabilities (words, numbers, diagrams) | No |
| 1. Allows the patient to select a way of viewing probabilities (words, numbers, diagrams) | No |
| 1. Allow patient to view probabilities based on their own situation (e.g. age) | No |
| 1. Place probabilities in context of other events | No |
| 1. Use both positive and negative frames (e.g. showing both survival and death rates) | No |
| **Include methods for clarifying and expressing patients’ values?** | |
| 1. Describe the procedure and outcomes to help patients imagine what it is like to experience their physical, emotional, social effects | Yes |
| 1. Ask patients to consider which positive and negative features matter most | Yes |
| 1. Suggest ways for patients to share what matters most with others | Yes |
| **Include structured guidance in deliberation and communication?** | |
| 1. Provide steps to make a decision | Yes |
| 1. Suggest ways to talk about the decision with a health professional | Yes |
| 1. Include tools (worksheet, question list) to discuss options with others | Yes |
| **Domain 2. Development Process: does the patient decision aid…** | |
| **Present information in a balanced manner?** | |
| 1. Able to compare positive/negative features of options? | Yes |
| 1. Shows negative/positive features with equal detail (fonts, order, display of statistics) | Yes |
| **Have a systematic development process?** | |
| 1. Includes developers’ credentials/qualifications? | Yes |
| 1. Finds out what users (patients and practitioners) need to discuss options | Yes |
| 1. Has peer review by patient/professional experts not involved in the development and field testing | Yes |
| 1. Is field tested with users (patients facing the decision; practitioners presenting options) | Not yet |
| *The field tests with users (patients, practitioners) show the patient decision aid is:* | |
| 1. Acceptable | NA |
| 1. Balanced for undecided patients | NA |
| 1. Understood by those with limited reading skills | NA |
| **Use up to date scientific evidence that is cited in a reference section or technical document?** | |
| 1. Provides references to evidence used | Yes |
| 1. Report steps to find, appraise, summarize evidence | Yes |
| 1. Report date of last update | Yes |
| 1. Report how often patient decision aid is updated | Yes |
| 1. Describe quality of scientific evidence (including lack of evidence) | Yes |
| 1. Uses evidence from studies of patients similar to those of target audience | Yes |
| **Disclose conflicts of interest?** | |
| 1. Report source of funding to develop and distribute the patient decision aid | Yes |
| 1. Report whether authors of their affiliations stand to gain or lose by choices patients make after using the patient decision aid | Yes |
| **Use plain language?** | |
| 1. Is written at a level that can be understood by the majority of patients in the target group | Yes |
| 1. Is written at a grade 8 equivalent level or less according to readability score (SMOG or FRY) | Yes |
| 1. Provides ways to help patients understand information other than reading (audio, video, in-person discussion) | Yes |
| *Meet additional criteria if the patient decision aid is Internet based* | |
| 1. Provide a step-by-step way to move through the web pages | Yes |
| 1. Allow patients to search for key words | No |
| 1. Provide feedback on personal health information that is entered into the patient decision aid | NA |
| 1. Provides security for personal health information entered into the decision aid | Yes |
| 1. Make it easy for patients to return to the decision aid after linking to other web pages | Yes |
| 1. Permit printing as a single document | Yes |
| *Meet additional criteria if stories are used in the patient decision aid* | |
| 1. Use stories that represent a range of positive and negative experiences | NA |
| 1. Reports if there was a financial or other reason why patients decided to share their story | NA |
| 1. State in an accessible document that the patient gave informed consent to use their stories | NA |
| **Domain 3. Effectiveness: Does the patient decision aid ensure decision making is informed and values based?** | |
| **Decision processes leading to decision quality. The patient decision aid helps patients to…** | |
| 1. Recognize a decision needs to be made | NA |
| 1. Know options and their features | NA |
| 1. Understand that values affect decision | NA |
| 1. Be clear about option features that matter most | NA |
| 1. Discuss values with their practitioner | NA |
| 1. Become involved in preferred ways | NA |
| **Decision quality. The patient decision aid…** | |
| 1. Improves the match between the chosen option and the features that matter most to the informed patients | NA |
